# Healthcare resource utilization and costs before and after long-acting injectable antipsychotic initiation in commercially insured young adults with schizophrenia

**DOI:** 10.1186/s12888-022-03895-2

**Published:** 2022-04-09

**Authors:** Alex Z. Fu, Jacqueline A. Pesa, Susan Lakey, Carmela Benson

**Affiliations:** 1 Janssen Scientific Affairs, LLC, Titusville, NJ USA; 2 Georgetown University Medical Center, Washington, DC USA

**Keywords:** Schizophrenia, Long acting injectables, Hospitalization, Relapse, Healthcare resource utilization and costs

## Abstract

**Background:**

Long-acting injectable (LAI) antipsychotics use is associated with improved adherence which can reduce the rate of relapse, hospitalization, and associated costs in patients with schizophrenia. Young adults could be at higher risk of poor adherence, hence use of LAI in this population may offer a benefit but the evidence is limited. This study aimed to compare clinical and economic outcomes before and after the initiation of LAI antipsychotics in commercially insured young adults (18-35 years of age) with schizophrenia.

**Methods:**

A retrospective claims data study was conducted using the data from the IBM MarketScan® Commercial Claims and Encounters (CCAE) Database. Patients with a continuous enrollment of at least 1-year before and 1-year after the first observed schizophrenia diagnosis (index date) and with the use of ≥1 typical or atypical LAI antipsychotic during the post-index follow-up period were included. A pre-post analysis was conducted to compare relapse rates, healthcare resource utilization, and costs before (from index date to LAI initiation) and after LAI initiation (to end of follow up).

**Results:**

A total of 2222 patients who initiated LAIs after an index schizophrenia diagnosis were identified. The per patient per month (PPPM) composite relapse event rate (0.109 pre-LAI to 0.073 post-LAI) and hospitalization rate (0.091 to 0.058), all-cause inpatient visits (0.231 to 0.119), and length of stay (2.694 to 1.092 days) significantly decreased from before LAI initiation to after LAI initiation with similar trends seen for mental health and schizophrenia-related measures (all significant; *P* < 0.0001). All-cause total costs ($4898 to $3078 PPPM) were also decreased after LAI initiation, with similar trends seen for mental health and schizophrenia-related costs (all significant; *P* < 0.0001). Although medication costs were higher post-LAI period ($311 to $542 PPPM), the cost increase was substantially offset by the decreased costs associated with total healthcare costs.

**Conclusions:**

Treatment with LAI antipsychotics was associated with a decrease in relapse event rate, healthcare resource utilization, and costs after LAI initiation compared to before LAI initiation in commercially insured young adults with schizophrenia. Treatment with LAIs in young adults with schizophrenia is potentially associated with significant cost savings to commercial payers.

## Background

Schizophrenia spectrum disorders are among the most disabling and economically catastrophic medical disorders; 80 to 90% of patients with schizophrenia are unemployed [1, 2], 20% are homeless [2] and 17% are incarcerated [3, 4]. The disease affects up to 1% of the United States (US) population [5] and is characterized by recurrent episodes of debilitating illness symptoms like psychosis, delusions, and impairments in cognitive and social functioning [6, 7], alternating with periods of full or partial remission. Moreover, schizophrenia manifests in young adulthood, making the burden of illness over time substantial. A prevalence-based study in the US population has reported the overall annual costs attributable to schizophrenia are $155.7 billion, including $9.3 billion in direct costs and $117.3 billion in indirect costs [8].

Antipsychotic medications are an effective first-line and maintenance treatment for schizophrenia [9, 10]; however, treatment compliance can be a challenge for many reasons. Features of the disease such as low insight and paranoia, combined with cognitive decline that develops over recurrent episodes often lead to treatment non-compliance, episode recurrence and disease progression. Treatment in the initial stages of the disorder is crucial for reducing clinical symptoms and functional impairment, preserving neurocognition, and improving long-term outcomes among young patients with recent-onset psychotic disorders [11–13]. However, treatment compliance among early-phase patients has been found to be low, with 42% of first-episode patients discontinuing their oral antipsychotic medication during the first year of treatment [14]. Compared to oral antipsychotics, improved treatment compliance and the associated clinical and economic outcomes have been found among patients with schizophrenia who use long-acting injectable (LAI) antipsychotics [15–19], administered at twice- monthly or longer intervals.

The American Psychiatric Association [6], The National Council for Mental Wellbeing [20], and the 2019–2020 Florida Best Practice Psychotherapeutic Medication Guidelines [21] have laid guideline recommendations for the use of LAI antipsychotics. Supported by a substantial body of literature, these guideline recommendations encourage LAI antipsychotics if patients prefer such treatment or if they have a history of poor or uncertain adherence. Similarly, in the case of newly diagnosed patients, patients with a recent relapse or those transitioning from in-patient care or incarceration, LAI should be used as an early treatment option and not only after multiple negative outcomes such as failed oral medications, multiple relapses, or hospitalizations [6, 20, 21].

Despite available evidence of poor compliance with oral antipsychotics [14], many clinicians do not consider the use of LAI antipsychotics in the early stages of patients with schizophrenia, assuming patients would prefer oral treatments [17]. Generally, use of LAIs has been reserved for very sick, hard-to-treat patients with schizophrenia. However, this practice may have possibly changed in the last decade driven by the introduction of several LAI’s in the US since 2010 to date, rapid advocacy, and the recommendations from various guidelines on the earlier use of LAI for patients diagnosed with schizophrenia [6, 20–22].

There is a limited real-world data available focused on outcomes of younger, commercially insured patients with schizophrenia treated with LAI antipsychotics. Moreover, since 2010, the Affordable Care Act (ACA) has required private health plans to extend dependent coverage to adults up to age 26 [23]. The ACA’s dependent coverage expansion was associated with a higher likelihood that psychosis treatment for young adults was covered by private insurance and a lower likelihood that they were covered by public insurance [23]. Early use of LAIs in younger patients may help prevent disease deterioration at the early stage of the disease. Therefore, there is a need to study real-world outcomes based on data available on young adult patients with schizophrenia in private commercial insurance databases. The aim of this study is to compare clinical and economic outcomes before and after treatment with an LAI antipsychotic in commercially insured young adults (18-35 years of age at first observed schizophrenia diagnosis) with schizophrenia. Demographics and clinical characteristics of young adults with schizophrenia treated with LAI antipsychotics are reported. Relapse rates, healthcare resource utilization (HCRU), and costs are compared before and after the initiation of an LAI antipsychotic in this group of patients.

## Methods

### Data sources and patient selection

This retrospective, observational cohort study used administrative medical and pharmacy claims data derived from the IBM MarketScan® Commercial Claims and Encounters (CCAE) Database. The CCAE database is a fully adjudicated, paid medical and pharmacy insurance claims database of approximately 138 million unique de-identified persons that include active employees, early retirees, COBRA continuers, and their dependents insured by employer-sponsored plans. The database contains inpatient admission records, outpatient services, prescription drugs, enrollment status, and costs of medical services and drugs. It captures person-specific clinical utilization, cost, and enrollment across inpatient, outpatient, prescription drug, and carve-out services.

Patients with ≥1 inpatient or ≥ 2 outpatient claims with a diagnosis code for schizophrenia. (ICD-9-CM: 295.xx; ICD-10-CM: F20.x, F21.x, F25.x) during the intake period (January 1, 2008, to December 31, 2019) were identified; the date of first observed diagnosis served as the index date. Patients aged 18-35 years at index date, with a continuous enrollment of at least 1 year before and 1 year after the index date and with the use of ≥1 typical (first-generation, [fluphenazine, haloperidol]) or atypical (second-generation, [aripiprazole, aripiprazole lauroxil, olanzapine pamoate, paliperidone palmitate, risperidone microsphere, risperidone subcutaneous]) LAI antipsychotic during the post-index follow-up period were included. The 1-year pre-index period was used as the baseline to define patient characteristics.

### Outcomes

The outcomes of interest included demographics and clinical characteristics of study patients, time from index date to LAI initiation, time from LAI initiation to relapse, relapse rates during the follow-up period (compared between pre−/post- LAI initiation period) and average healthcare resource utilization and cost during the follow-up period (compared between pre−/post- LAI initiation period).

Demographic characteristics for the cohort included age, gender, and region; clinical characteristics included baseline antipsychotic treatment, most common mental and physical comorbidities, baseline medications, and LAI initiation (provider type and setting).

Inpatient admissions and emergency department (ED) visits served as proxies for relapse and are reported both separately and as part of a composite measure of relapse (defined as any inpatient admission or ED visit with a diagnosis of schizophrenia as the primary or secondary diagnosis). The relapse rate is presented as per patient per month (PPPM).

HCRU outcome measures included number of inpatient admissions, length of stay, number of ED visits, number of outpatient visits, and number of prescription medications filled; HCRU costs were payment amounts associated with all utilization measures. For consistency across HCRU outcomes assessed, all measures and associated costs are presented as PPPM and separately for all-cause, mental health-related, and schizophrenia-related components.

### Statistical analyses

Demographic and clinical characteristics from the baseline period are presented descriptively. Means and standard deviations were reported for continuous variables and numbers and percentages were reported for categorical variables. Kaplan-Meier estimator was used to examine the time from index date to LAI initiation, as well as time from LAI initiation to first relapse with right censoring for end of continuous enrollment or end of data cut.

The PPPM relapse rate was calculated from the index date to the end of the follow-up period and was expressed as the rate per patient per month. The relapse rate was calculated as “number of events within the specific period” divided by “duration of the specific period in months” for a given patient. HCRU and costs were calculated separately for the 2 time periods: index date to LAI initiation and LAI initiation to the end of follow-up.

A pre-post analysis was conducted using McNemar’s test to compare relapse rates and paired t-tests to compare HCRU and costs before (from index date to LAI initiation) and after LAI initiation (to end of follow-up). A *p*-value of < 0.05 was considered statistically significant. All data analyses were conducted using SAS Enterprise Guide 8 (SAS Institute, Cary, NC).

## Results

After applying inclusion criteria, a total of 2222 patients who initiated LAIs after an index schizophrenia diagnosis were identified and included in the analysis. The post-index follow-up period ranged from 1 to 10.9 years with an average of 2.7 years.

### Demographics and clinical characteristics of patients

The mean age of the patients was 22.9 years and the majority of the patients (82.4%) were in the 18-25 age group (Table 1). The study population included a higher proportion of men (70.4%) than women (29.6%). Considering the geographic/regions distribution, the South region (38.6%) had the highest representation of patients followed by the Midwest (23.2%), Northeast (19.3%), and West (18.3%). 61.7% of the cohort had been treated with antipsychotics prior to their index schizophrenia diagnosis (index date) and 38.3% reported no evidence of prior treatment with antipsychotics in the baseline period. During the baseline period, 49.2% of patients had claims for oral atypical antipsychotics, followed by typical LAIs (9.3%), oral typical antipsychotics (8.5%), and atypical LAIs (6.9%). Patients were also frequently prescribed mood stabilizers (37.8%), anxiolytics (27.5%), opioids (19.5%), and antidepressants (7.0%) over the baseline period.

**Table 1:** Characteristics of study patients treated with LAIs

Characteristic	LAI patients (***N*** = 2222)
**Age at index, mean (SD)**	**22.9 (4.0)**
18-25, n (%)	1832 (82.4)
26-35, n (%)	390 (17.6)
**Gender, n, (%)**	
Male	1565 (70.4)
Female	657 (29.6)
**Region, n (%)**	
Northeast	428 (19.3)
Midwest	516 (23.2)
South	858 (38.6)
West	407 (18.3)
Other/Unknown	13 (0.6)
**Baseline antipsychotic treatment, n (%)**	
Treated	1370 (61.7)
Untreated	852 (38.3)
**DSM-V mental illness comorbidities in 12-month pre-index period, n (%)**	
Substance-related and addictive disorders	816 (36.7)
Anxiety disorders	746 (33.6)
Bipolar and related disorders	700 (31.5)
Depressive disorders	552 (24.8)
Neurodevelopmental disorders	413 (18.6)
Trauma- and stressor-related disorders	357 (16.1)
Other conditions that may be a focus of clinical attention	329 (14.8)
Sleep-wake disorders	246 (11.1)
Other mental disorders	201 (9.0)
Disruptive, impulse-control, and conduct disorders	158 (7.1)
Personality disorders	128 (5.8)
**Physical health comorbidities in 12-month pre-index period, n, (%)**	
Other neurological disorders	382 (17.2)
Hypertension, uncomplicated	170 (7.7)
Obesity	165 (7.4)
Fluid and electrolyte disorders	151 (6.8)
**Baseline medications**	
Antipsychotics, n (%)	
LAI atypical antipsychotics	154 (6.9)
LAI typical antipsychotics	206 (9.3)
Oral atypical antipsychotics	1094 (49.2)
Oral typical antipsychotics	188 (8.5)
Antidepressants, n (%)	155 (7.0)
Anxiolytics, n (%)	612 (27.5)
Mood stabilizers, n (%)	841 (37.8)
Opioids, n (%)	434 (19.5)
**LAI initiation**	
Type of providers at LAI initiation, n, (%)	
Psychiatrist	708 (31.9)
Primary care	68 (3.1)
Others	1446 (65.1)
Healthcare setting at LAI initiation, n, (%)	
Inpatient stay	1560 (70.2)
ER visit	120 (5.4)
Others (e.g., Outpatient, SNF, OOV)	542 (24.4)

*DSM-5* Diagnostic and Statistical Manual of Mental Disorders, 5th Edition, *ER* emergency room, *LAI* long-acting injectable, *OOV* other office visit, *SD* standard deviation, *SNF* skilled nursing facility

The most common mental health comorbidities in the baseline period included substance abuse (36.7%), anxiety (33.6%), bipolar (31.5%), and depressive (24.8%) disorders. The diagnosed physical health comorbidities were relatively low, with the most common being other neurological disorders (17.2%) and uncomplicated hypertension (7.7%).

Among total patients, 70.2% of LAI initiation occurred during an inpatient stay, 24.4% during other visit (e.g., outpatient visit, other office visit, skilled nursing facility visit), and 5.4% during an emergency room (ER) visit.

### LAI initiation and relapse rate

**Fig. 1:**
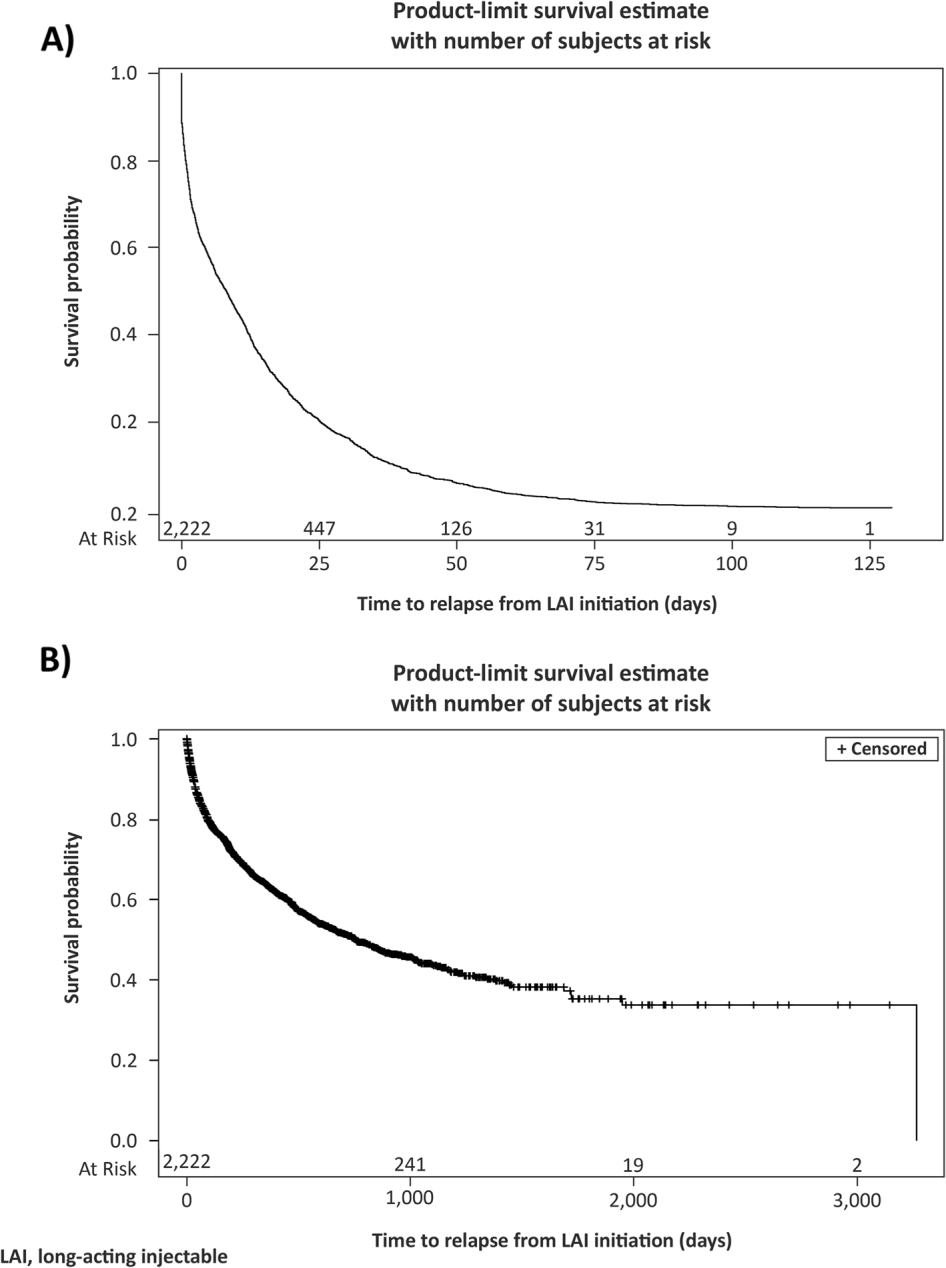
Time from (**A**) index date to LAI initiation and (**B**) LAI initiation to relapse

The average time from index date to LAI initiation was 433.1 days (median 243 days), and from LAI initiation to the end of follow-up was 744.0 days (median 630 days) (Fig. 1A). The median time from LAI initiation to relapse was 759 days (Fig. 1B).

The PPPM composite relapse event rate decreased significantly from 0.109 prior to LAI initiation to 0.073 following LAI initiation (*P* < 0.0001) (Table 2). The hospitalization rate also decreased significantly from 0.091 prior to LAI initiation to 0.058 following LAI initiation (*P* < 0.0001). ER visit rate slightly decreased from the pre-initiation period to post-initiation; however, the difference was not significant (*P* = 0.2552).

**Table 2:** Average relapse rate during follow-up period

	Period from index date to the end of F/U, among patients who initiated LAI after index date (***N*** = 2222)		
	From index date to LAI initiation	From LAI initiation to end of F/U	***P*** value
Relapse rate (PPPM)^a,b^	0.109	0.073	< 0.0001
Hospitalization rate (PPPM)	0.091	0.058	< 0.0001
ER rate (PPPM)	0.017	0.015	0.2552

*ER* emergency room, *F/U* follow-up, *LAI* long-acting injectable, *PPPM* per patient per month

^a^Relapse is defined as hospitalization or ER visits with a diagnosis of schizophrenia as primary or secondary diagnosis, assessed from index date to the end of follow-up (F/U) period

^b^Rate in PPPM is calculated as “number of events within the specific period” divided by “duration of the specific period in months” for a given patient

### Health care resource utilization and costs

All-cause inpatient visits reduced significantly from 0.231 PPPM prior to LAI initiation to 0.119 following LAI initiation, with similar trends seen for mental health (0.201 to 0.098) and schizophrenia-related (0.180 to 0.070) measures (all *P < 0.0001*). All-cause length of stay decreased significantly from 2.694 to 1.092 days, with similar trends seen for mental health (2.600 to 1.060 days) and schizophrenia-related (2.576 to 0.954 days) measures (all *P* < 0.0001) (Table 3).

**Table 3:** Average healthcare utilization and costs (PPPM) during follow-up period

	Period from index date to the end of F/U, among patients who initiated LAI after index date (***N*** = 2222)		
	From index date to LAI initiation	From LAI initiation to end of F/U	***P*** value
**All-cause HCRU and costs**			
Inpatient admission			
Number of admissions	0.231	0.119	< 0.0001
Length of stay (days)	2.694	1.092	< 0.0001
Costs	3562.78	1491.25	< 0.0001
ER visit			
Number of visits	0.090	0.102	< 0.0001
Costs	169.66	184.93	0.0002
Other visit (e.g., Outpatient, SNF, OOV)			
Number of visits	4.459	4.687	< 0.0001
Costs	854.41	858.55	< 0.0001
Prescription medications			
Number of prescriptions	2.044	2.345	< 0.0001
Costs	310.94	542.19	< 0.0001
All-cause total medical costs (without medications)	4586.85	2534.73	< 0.0001
All-cause total costs	4897.79	3077.92	< 0.0001
**Mental health-related HCRU and costs**			
Inpatient admission			
Number of admissions	0.201	0.098	< 0.0001
Length of stay (days)	2.600	1.060	< 0.0001
Costs	2582.16	1001.24	< 0.0001
ER visit			
Number of visits	0.050	0.049	0.7425
Costs	75.48	78.07	0.7465
Other visit (e.g., Outpatient, SNF, OOV))			
Number of visits	2.050	1.980	0.5212
Costs	485.50	458.06	0.5635
Prescription medications			
Number of prescriptions	0.990	1.140	< 0.0001
Costs	140.66	325.64	< 0.0001
Total medical costs (without medications)	3143.14	1537.37	< 0.0001
Total costs	3283.60	1863.01	< 0.0001
**Schizophrenia-related HCRU and costs**			
Inpatient admission			
Number of admissions	0.180	0.070	< 0.0001
Length of stay (days)	2.576	0.954	< 0.0001
Costs	2525.58	739.57	< 0.0001
ER visit			
Number of visits	0.026	0.018	0.0045
Costs	32.47	27.82	0.3096
Other visit (e.g., Outpatient, SNF, OOV)			
Number of visits	1.190	1.120	0.3075
Costs	261.16	209.94	0.0577
Prescription medications			
Number of prescriptions	0.471	0.580	< 0.0001
Costs	117.91	297.55	< 0.0001
Total medical costs (without medications)	2819.21	977.34	< 0.0001
Total costs	2937.12	1274.88	< 0.0001

*ER* emergency room, *HCRU* healthcare resource utilization, *F/U* follow-up, *LAI* long-acting injectable, *OOV* other office visit, *PPPM* per patient per month, *SNF* skilled nursing facility

Rate in PPPM is calculated as “number of events within the specific period” divided by “duration of the specific period in months” for a given patient

PPPM cost is calculated as “total costs within specific period” divided by “duration of specific period in months” for a given patient

SNF and OOV costs are included in the total cost

Healthcare costs are adjusted for inflation using the medical care component of the US Consumer Price Index and reported in 2020 US dollars

All-cause direct medical cost (excluding medication), medication cost, and total costs (with medication) were $4587, $311, and $4898 PPPM before and $2535, $542, and $3078 PPPM after LAI initiation, respectively (all significant; *P* < 0.0001) (Table 3).

Mental health-related medical cost (excluding medication), medication cost, and total costs (with medication) were $3143, $141, and $3284 PPPM before and $1537, $326, and $1863 PPPM after LAI initiation (all significant; *P* < 0.0001) (Table 3).

Schizophrenia-related medical cost (excluding medication), medication cost, and total costs (with medication) were $2819, $118, and $2937 PPPM before and $977, $298, and $1275 PPPM after LAI initiation (all significant; *P* < 0.0001) (Table 3). Although medication costs increased post-LAI period, the cost increase was substantially offset by the decreased costs associated with total healthcare costs.

## Discussion

This retrospective claims-based cohort study assessed demographic and clinical characteristics, improvement in relapse and hospitalization rates, HCRU, and associated costs before and after the initiation of LAI antipsychotics among commercially insured young adults with schizophrenia (18-35 years at index date) in the US population. There is a paucity of real-world data regarding outcomes associated with the usage of LAIs in younger, commercially insured patients with schizophrenia.

The use of an antipsychotic medication remains the first-line treatment for patients with schizophrenia [24]. However, patients with early-stage schizophrenia are often non-adherent to oral antipsychotic medication and need continuous and long-term treatment [25–27]. LAI antipsychotics offer a treatment option for such patients to improve daily adherence with comparable efficacy and tolerability [28]. A recent study evaluated the real-world effectiveness of LAI antipsychotics over oral antipsychotics in a younger age group (18-35 years) of adult US patients with schizophrenia based on the IBM Watson Health MarketScan Medicaid Database [29]. The study demonstrated that younger adults taking LAI antipsychotics have increased treatment adherence, lower concomitant use of psychiatric medications, and lower polypharmacy compared to treatment with oral antipsychotics during a 12-month follow-up period. Another study by Pilon et al. also demonstrated that the use of LAIs led to better adherence and prevented extensive use of other antipsychotic medications compared to oral antipsychotics among younger patients with schizophrenia (18-25 years) [30]. In addition, early initiation of LAI is reported to be associated with better treatment outcomes and reduced costs in managing patients with schizophrenia. A recently conducted retrospective cohort analysis using claims data from Truven Health Analytics MarketScan Commercial, Medicaid, and Medicare supplemental databases reported that LAI initiation within 1 year of a new schizophrenia episode led to lower hospitalization rates and healthcare costs compared with LAI initiation of more than 1 year after a new episode [31].

Recent evidence has suggested that the occurrence of relapses led to reduced or delayed antipsychotic treatment response rates in schizophrenia patients [32]. It was reported that approximately half of the patients failed to achieve a 50% response rate (≥50% reduction in the brief psychiatric rating scale [BPRS] total score) to antipsychotic treatments in the second episode compared to the majority of patients (more than 90%) achieving 50% response rate during the first episode. Relapses are also significantly associated with the reduced quality of life in patients with schizophrenia [33]. Hence it is important to reduce relapse rates, particularly in younger patients. We observed a significant decrease of PPPM composite relapse rate (0.109 to 0.073), and hospitalization rate (0.901 to 0.058) from before LAI initiation to after LAI initiation in our study.

Several other published studies have also reported reduced relapse rate and hospitalization associated with the use of LAI in patients with schizophrenia though these studies did not report data, particularly for the 18-35 years-of-age group patients. In a meta-analysis of 10 randomized long-term trials, LAI formulations were reported to significantly reduce relapses with relative and absolute risk reductions of 30 and 10%, respectively, when compared with oral antipsychotics [34]. A meta-analysis based on 42 prospective and retrospective cohort studies demonstrated that LAIs were superior to oral antipsychotics regarding hospitalization rate with reduced hospitalization rates in patients receiving LAIs (rate ratio = 0.85, 95% confidence interval [CI] = 0.78-0.93, *P* < 0.001) [35]. Another meta-analysis which included 25 mirror-image studies from 28 countries that followed 5940 patients with schizophrenia for ≥12 months reported that LAIs showed strong superiority over oral antipsychotics in preventing hospitalization (risk ratio = 0.43; 95% CI, 0.35-0.53; *P* < 0.001) and in decreasing the number of hospitalizations (rate ratio = 0.38; 95% CI, 0.28-0.51; *P* < 0.001) [36]. Several other studies have also reported the superiority of LAIs in preventing hospitalization and relapses in patients with schizophrenia compared to oral antipsychotics [37, 38].

In our analysis, we observed a significant reduction in all-cause inpatient visits and all-cause length of hospital stay from pre-LAI initiation to post-LAI initiation periods. Similar trends were observed for mental health-related and schizophrenia-related inpatient admission. Our findings are consistent with other published studies reporting a reduction in the length of hospital stay post LAI-treatment [29, 39–41]; although the duration of stay was considerably shorter in our study which included younger patients (mean age ~ 23 years). However, the rates if converted to per patient per year are comparable to other studies [42]. A recent retrospective study of 210 patients with schizophrenia (mean age 34.2 years) based on the electronic medical records from Atrium Health in a US integrated healthcare system demonstrated a significant decrease in all-cause inpatient visits (67.6 to 22.4%, *P* < 0.001) and mean length of inpatient stay (14.2 ± 16.8 to 4.4 ± 13.2 days, *P* < 0.001) from the period before LAI initiation to 12 months after LAI initiation. Similar trends in mental health-related inpatient visits (67.6 to 22.4%, *P* < 0.001) and schizophrenia-related inpatient visits (61.4 to 20.5%, *P* < 0.001) were reported [40]. Another claim data study among US-based young adult Medicaid patients with schizophrenia reported 37% reduced all-cause inpatient admissions (odds ratio [OR], 0.63; 95% CI, 0.53–0.74) after initiating LAI compared to oral atypical antipsychotics cohort [29]. A retrospective claim-based study of 1272 South Korean patients with schizophrenia using the Health Insurance Review and Assessment Service (HIRA) database demonstrated a significant reduction in the proportion of patients’ admission to hospital (35.9 to 19.3%, *P* < 0.0001) from pre-LAI initiation to post-LAI initiation periods [41]. However, it should be acknowledged that clinical factors such as disease severity, presence of comorbidities, and treatment received at baseline or within 48 h during hospitalization combined with discharge processes can be determinants of hospitalization rates, length of stay and the associated costs [42–44].

We observed a significant reduction in all-cause direct medical cost ($4587 to $2535) and total costs ($4898 to $3078) PPPM from pre-LAI initiation and post-LAI initiation in young adults with schizophrenia, with similar trends for mental health-related and schizophrenia-related medical cost and total costs. A US-based claim data study using IBM Watson Health MarketScan Medicaid Database reported significantly lower total costs (difference in PMPM mean costs = $242 [− 62, 469]) and medical costs (difference in per member per month [PMPM] mean costs = $364 [− 673, − 184]) among young adults of 18-35 years taking LAI vs oral antipsychotics [29]. Further, a MarketScan Multi-state Medicaid database study of 2302 adult patients with schizophrenia found reduced monthly inpatient ($US4007 vs. 8769, *P* < 0.001) and ER visits costs ($682 vs. 891, *P* < 0.001), but increased monthly medication costs ($10,713 vs. $655, *p* < 0.001) for patients using LAI compared to the oral cohort over the 12-month post-index period [45]. Several other studies have also reported decreased medical costs for patients taking LAIs [30, 41].

The present analysis has several limitations. Results from this study may not be generalizable to patients with other types of health insurance coverage, such as those with public insurance (e.g., Medicaid) or those without health insurance coverage. Claims data are intended for billing purposes; hence diagnosis is subject to coding inaccuracies, resulting in potential misclassification bias. The 18–35year old patients may best approximate newly diagnosed patients or new psychotic episodes, but the claims databases do not include a clinical endpoint to confirm this. The regression to the mean is a potential limitation for pre-post study designs. The observed favorable impact of LAIs is likely also biased due to the established tolerability of the prior oral drug exposure, enhancing the LAI's apparent effectiveness, and impact on health care resource utilization and cost. Patients were followed for different post-index follow-up periods as per the availability of data for individual patients. Follow-up for consistent and specific time frames for different patients might have provided more consistent patient experiences to make better comparisons, e.g., a small percentage of patients may have remitted and no longer needed treatment [46, 47]. Our data do not address the adverse effects of LAIs, which must be weighed against the benefit. Additionally, the prescription dispensing data do not necessarily reflect the actual use of medication and could potentially result in overestimation of utilization. Furthermore, the claims database does not support the plausibility of ascertaining the reasons for the end of follow-up, which might include remission, discontinuation due to adverse events, or withdrawal of consent for treatment. Nonetheless, LAI antipsychotic treatment, along with other strategies as suggested by Curto et al., is an option to support effective long-term treatment adherence [48].

## Conclusions

In summary, the use of LAIs was associated with decreased relapse event rate in the period after LAI initiation as compared with the period before LAI initiation in commercially insured young adults with schizophrenia. In addition, LAI use was associated with decreases in HCRU and costs in the period after LAI initiation as compared with the period before LAI initiation. The increased medication cost post LAI-initiation period was substantially offset by decreased total healthcare costs. The LAI antipsychotic treatment for young adults with schizophrenia is potentially associated with significant cost savings to commercial payers.

## Data Availability

The current study uses administrative medical and pharmacy claims data derived from the IBM MarketScan® Commercial Claims and Encounters (CCAE) Database. The data is available on a paid subscription basis.

## Abbreviations

CCAE: Commercial Claims and Encounters
ED: Emergency department
ER: Emergency room
HCRU: Healthcare resource utilization
HIRA: Health Insurance Review and Assessment Service
LAI: Long-acting injectable
OFV: Office visit
PPPM: Per patient per month
SNF: Skilled nursing facility
US: United States
